# Sunlight-Active BiOI Photocatalyst as an Efficient Adsorbent for the Removal of Organic Dyes and Antibiotics from Aqueous Solutions

**DOI:** 10.3390/molecules26185624

**Published:** 2021-09-16

**Authors:** Teerapong Narenuch, Teeradech Senasu, Tammanoon Chankhanittha, Suwat Nanan

**Affiliations:** Materials Chemistry Research Center, Center of Excellence for Innovation in Chemistry (PERCH-CIC), Department of Chemistry, Faculty of Science, Khon Kaen University, Khon Kaen 40002, Thailand; teerapong_narenuch@kkumail.com (T.N.); teera_sen@kkumail.com (T.S.); tammanoon_chan@hotmail.com (T.C.)

**Keywords:** BiOI, photodegradation, antibiotics, organic dyes, solar light

## Abstract

A bismuth oxyiodide (BiOI) photocatalyst with excellent sunlight-driven performance was synthesized by a solvothermal route without the addition of surfactants or capping agents. The prepared photocatalyst exhibited a tetragonal phase with an energy band gap of 2.15 eV. The efficiency of the photocatalyst was elucidated by monitoring the photodegradation of organic dyes and antibiotics. The BiOI photocatalyst provided a 95% removal of norfloxacin (NOR) antibiotics under visible light illumination. Interestingly, the complete removal of Rhodamine B (RhB) dye was achieved after 80 min of natural sunlight irradiation. The photodegradation reaction followed the first-order reaction. Both photo-generated holes and electrons play vital roles in the photodegradation of the pollutant. The BiOI photocatalyst remains stable and still shows a high efficiency even after the fifth run. This confirms the great cycling ability and high structural stability of the photocatalyst. The prepared BiOI catalyst, with a high surface area of 118 m^2^ g^−1^, can act as an excellent adsorbent as well. The synergistic effect based on both adsorption and photocatalysis is a key factor in achieving a very high removal efficiency. The photoactivity under sunlight is higher than that observed under visible light, supporting the practical use of the BiOI photocatalyst for the removal of organic pollutants in wastewater through the utilization of abundant solar energy.

**Highlights:**

- A BiOI photocatalyst was successfully prepared using a solvothermal method.
- The complete photodegradation of RhB dye under solar light irradiation was achieved.
- The photodegradation reaction correlated well with the first-order kinetics model.
- The BiOI photocatalyst exhibited good structural stability and reusability after five cycles.

## 1. Introduction

It has been reported that considerable attention has been paid to contamination by organic pollutants in natural water [1]. The promotion of antibiotic-resistant bacteria and threats to ecosystem stability are two major problems. Fluoroquinolone-based antibiotics have been utilized for the treatment of bacterial-infectious diseases [2]. In addition, upon industrial development, a massive number of organic dyes have been discharged into natural water [3]. Some dyes have been shown to have carcinogenic and mutagenic properties. It has been reported that the large-scale usage of both organic dyes and antibiotic drugs causes a serious threat to the environment [2,3,4]. Thus, the removal of these pollutants from the ecosystem is urgently needed [5].

The removal of organic pollutants by some conventional treatments has been reported. The incomplete degradation of the pollutants together with the creation of secondary hazardous pollutants are the main drawbacks of this method [2]. Interestingly, photocatalytic treatment is a clean and eco-friendly process for the degradation of pollutants [5,6,7,8,9]. It is generally known that commercially available TiO_2_ is a UV-active photocatalyst (band gap of 3.2 eV). Thus, TiO_2_ shows a low performance under natural sunlight [10,11]. Interestingly, the development of visible-light-active photocatalysts is a challenging and promising research topic in the field of environmental protection. The removal of organic pollutants in wastewater can be performed very easily by the utilization of economical sunlight [12,13,14,15].

The synthesis of numerous Bi-based photocatalysts, showing an Aurivillius structure, including Bi_2_MoO_6_, BiVO_4_, and BiOX (X = Cl, I, and Br), has been investigated [16,17,18,19,20,21,22,23,24]. BiOI (*E*_g_ of 1.5–1.7 eV), which shows a high photocatalytic performance, has received great attention. This is due to its advantages of non-toxicity, great stability, and high sunlight-active photocatalytic performance [25]. The synthesis of BiOI by various techniques has been reported [26,27]. Practically, the solvothermal/hydrothermal route has been used for the preparation of many nanomaterials because of its advantages of simplicity and low expense. In addition, the fabrication of samples on a large scale can be performed using the solvothermal process [24].

This research focuses on the solvothermal fabrication of BiOI. The performance of the prepared photocatalyst was monitored by examining the photodegradation of organic dyes and antibiotics. The efficiency reached 71% and 95% rates of degradation of RhB dye and NOR antibiotics under visible light irradiation. The BiOI photocatalyst also exhibited the complete degradation of RhB dye under natural sunlight illumination, indicating its promising environmental protection properties for the detoxification of organic dyes and fluoroquinolone antibiotics in wastewater.

## 2. Experiment

### 2.1. Chemicals

The chemicals were utilized as received. The water was purified by a Millipore process (18.2 MΩ cm^−1^).

### 2.2. Fabrication of BiOI

The BiOI sample was fabricated using a solvothermal technique. Firstly, about 1.0878 g of Bi(NO_3_)_3_·5H_2_O was dissolved in 30 mL of ethylene glycol. Secondly, about 0.3883 g of KI was dissolved in 30 mL of ethylene glycol. Thirdly, the KI solution was added to the bismuth nitrate solution. The mixed solution was then put into an autoclave at 160 °C for 12 h. The precipitate was filtered, washed, and then dried at 80 °C for 4 h.

### 2.3. Characterization

The characterization of this substance has been reported elsewhere [3,6,7,8,9]. The crystal structure of the sample was investigated using powder X-ray diffraction (XRD) and monochromatic Cu K_α_ radiation. The morphological structure of the sample was elucidated by the scanning electron microscopic (SEM) method. The vibrational spectrum was recorded using an FT-IR spectrophotometer. The KBr pellet method was used for sample preparation. The UV-vis diffused reflectance spectrum was examined. The photoluminescence (PL) spectrum was determined using an excitation wavelength of 360 nm.

### 2.4. Photocatalytic Degradation of the Pollutants

The photocatalytic performance of the prepared BiOI catalyst was examined by determining the degradation of dyes and antibiotics under artificial visible light irradiation (a Panasonic daylight lamp, 15 W) and natural solar light illumination. Both Rhodamine B (RhB) and Rhodamine 6G (R6G) were used as models of organic dyes. In addition, both ofloxacin (OFL) and norfloxacin (NOR) were selected as representatives of fluoroquinolone-based antibiotics. The photocatalytic study can be seen in detail elsewhere [22,24]. The control experiment was performed by illuminating the pollutant solution without the addition of the prepared BiOI photocatalyst. The photocatalyst (50 mg) was suspended in a pollutant solution of 10 mg L^−1^ (total volume of 200 mL). About 5 mL of the sample was collected. The exact concentration of the pollutant was elucidated by measuring the absorbance at λ_max_ of 273, 286, 554, and 527 nm, respectively, for NOR, OFL, RhB, and R6G, using a UV-vis spectrophotometer.

The performance of the photocatalyst in the photodegradation of the pollutant was determined by Equation (1):

Performance (%) = (1 − C/C_0_) × 100% (1)

where C_0_ and C represent the initial concentration and the concentration of the pollutant solution after photo illumination.

The photoactivity of the BiOI photocatalyst can also be determined by calculating the photodegradation rate as follows:

dC/dt = −k_app_C (2)

ln(C_0_/C) = k_app_t (3)

where k_app_ represents the apparent rate constant of the first-order degradation reaction.

A trapping experiment was performed to study the active species involved in the degradation of RhB dye. The addition of *t*-butanol, NaN_3_, EDTA-2Na, and K_2_Cr_2_O_7_ to quench the hydroxyl radical, superoxide anion radical, hole, and electron, respectively, was carried out. Each scavenger (5 mM) was incorporated before the addition of the photocatalyst (50 mg). All other conditions were similar to those of the no-scavenger process.

The hydroxyl radicals were also monitored by the incorporation of the photocatalyst (50 mg) in a terephthalic acid (TA) solution (50 mL). The sample was then examined by a spectrofluorometric method using an excitation wavelength (λ_excitation_) of 315 nm.

The cycling ability of the prepared BiOI photocatalyst was also checked. After the first cycle of the photodegradation study, the photocatalyst was filtered, washed with ethanol and water, and dried at 80 °C for 6 h. The photocatalyst was then used for five successive cycles.

## 3. Results and Discussion

### 3.1. Characterization of the BiOI Photocatalyst

**Figure 1:**
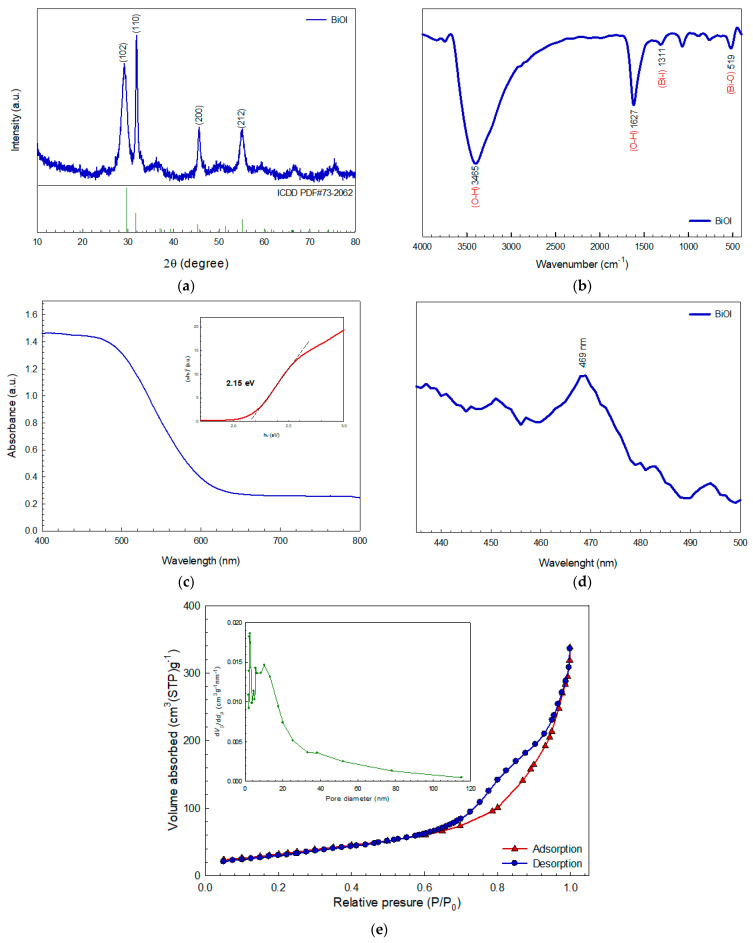
XRD pattern (**a**), FT-IR spectrum (**b**), UV-vis spectrum (**c**), PL spectrum (**d**), and nitrogen adsorption-desorption isotherm and BJH pore size distribution of the BiOI photocatalyst (**e**).

The XRD pattern of the prepared BiOI photocatalyst (Figure 1a) exhibited the peaks of a tetragonal structure at a 2θ of 28.96°, 31.73°, 45.43°, and 54.91°, corresponding to the reflection from the (102), (110), (200), and (212) lattice planes, respectively (JCPDS card no. 73-2060) [25]. No impurity peaks were observed. The crystallite size of the sample was determined from the width of the most intense peak (2θ = 31.73°) following the Scherrer equation. The BiOI photocatalyst showed the crystallite size of about 14.90 nm.

The vibrational spectrum of the BiOI (Figure 1b) provided vibrational bands at 519 cm^−1^ and 1311 cm^−1^ which were attributed to the Bi-O stretching mode and stretching vibrational mode of Bi-I bond, respectively [25]. The peak found at 1627 cm^−1^ was due to the flexural vibration of O-H in the adsorbed water [25]. In addition, the vibration band at 3465 cm^−1^ was assigned to the stretching vibration of water as well.

The UV-vis diffuse reflectance spectrum in Figure 1c provided the band gap energy (*E*_g_) of 2.15 eV following the Kubelka–Munk equation [22]. The sample provided promising visible-light-active photoactivity with an absorption edge of 577 nm. The charge carrier separation efficiency at the interface of the sample was determined from the photoluminescence spectrum (Figure 1d). The photocatalyst showed a PL peak at 469 nm with a high crystallinity [24].

In principle, it is well-accepted that the surface area and pore size of the sample strongly affects the adsorption ability and the photocatalytic performance of the resultant photocatalyst. Thus, the N_2_ adsorption-desorption isotherm was determined and then the BET surface area was elucidated. In addition, the BJH pore-size distribution of the sample was also investigated. The synthesized BiOI photocatalyst provided a high specific surface area of 118.19 m^2^ g^−1^, as shown in Figure 1e. The sample showed the type-IV isotherm, following the IUPAC classification, with the hysteresis loop of H_3_ indicating the existence of the mesopores in the prepared photocatalyst. The total pore volume of 0.45 cm^2^ g^−1^ and a pore size of 15.24 nm were reported. All in all, the very high surface area and meso-porous structure of the prepared BiOI photocatalyst are beneficial for the enhancement of adsorption ability, which in turn results in an increase in pollutant removal efficiency [3,28].

**Figure 2:**
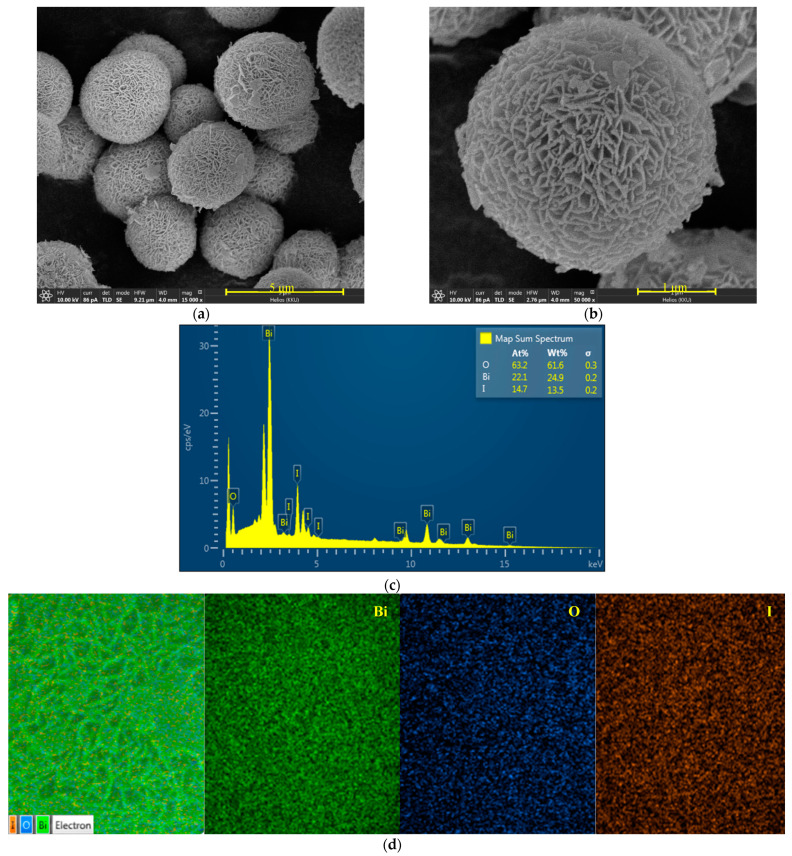
SEM micrographs (**a**,**b**); EDX spectrum (**c**); SEM micrograph of the mapping area; and EDX elementary mapping of Bi, O, and I obtained from the prepared BiOI photocatalyst (**d**).

The FE-SEM images of BiOI in Figure 2a,b show the spherical micro-structure of 3.41 μm, which has a flower-like morphology. It can be seen that each sphere was constructed using nanosheets as building blocks. The formation of the flower-like morphological structures is based on the connection of these nanosheets. The BiOI architectures were generated from the nanosheet arrays from the center to the surface of the spheres. In addition, it is well-accepted that BiOI shows a unique layered structure composed of [Bi_2_O_2_]^2+^ slabs interleaved by double slabs of iodine atoms [15]. It should be noted that ethylene glycol (EG), as a solvent, also plays an important role in controlling the final morphology of the sample. EG can act as a coordinating agent in the synthesis step, meaning that hierarchical BiOI structures can be achieved by inducing the self-assembly of the nanosheets to form hierarchical BiOI microspheres. The FESEM-EDX elemental mapping and EDX methods were used for investigating the elemental surface compositions and the distribution of the elements in the photocatalyst, respectively. The mapping micrographs (Figure 2d) confirmed the uniform dispersion of bismuth (Bi), oxygen (O), and iodine (I) in the prepared photocatalyst. Additionally, the weight% and atomic% ratios of the O:Bi:I elements were 61.6:24.9:13.5 and 63.2:22.1:14.7, respectively (Figure 2c).

### 3.2. Photodegradation Study

The photoactivity of the prepared BiOI photocatalyst in the photodegradation of dyes and antibiotics under visible light and natural solar light irradiation was investigated.

#### 3.2.1. Photodegradation of Pollutants

**Figure 3:**
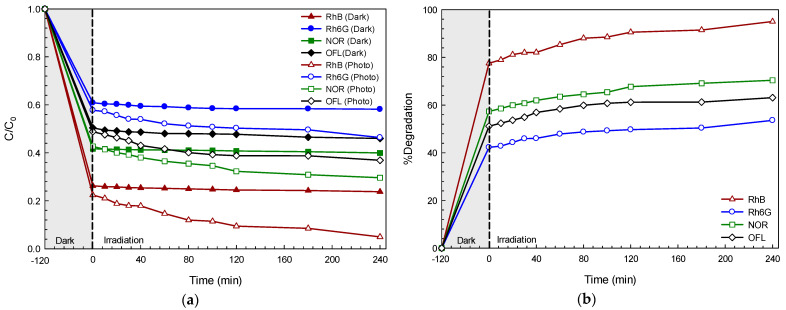
Plots of pollutant concentration (C/C_0_) vs. time in the presence of BiOI photocatalyst under visible light irradiation (**a**) and the corresponding photocatalytic efficiency (**b**).

**Figure 4:**
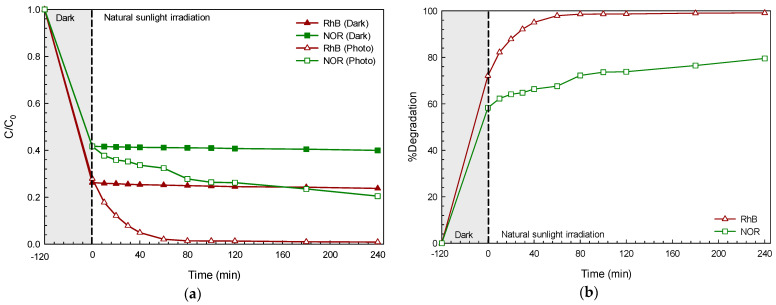
Plots of pollutant concentration (C/C_0_) vs. time in the presence of BiOI photocatalyst under natural sunlight irradiation (**a**) and the corresponding photocatalytic efficiency (**b**).

On examining the photodegradation of each pollutant under visible light illumination, a decrease in pollutant concentration with time was observed (Figure 3a). No photolysis of the pollutant was detected. In the present study, the performance of the BiOI with regard to the adsorption (in dark conditions) and photocatalytic degradation (under photo illumination) of the dyes and antibiotics was examined. The removal of these pollutants via the adsorption process was in the range of 41% (Rh6G) to about 76% (RhB) after 240 min. In the presence of the BiOI photocatalyst and light, the lowering of the pollutant concentration (C/C_0_) with time was continuously observed after 240 min. The enhanced photoactivity reached 71% and 95% in the degradation of the NOR antibiotic and RhB dye, respectively (Figure 3b). The photodegradation reaction fit very well with the first-order reaction [3]. In addition, solar light-responsive photocatalytic performances of 80% and 99% were detected in the degradation of NOR and RhB, respectively (Figure 4). A removal of the pollutant via the adsorption process in the rage of 60% (NOR) to 76% (RhB) was reported. This may be due to the very high surface area (about 118 m^2^ g^−1^) of the prepared BiOI, which can act as an excellent adsorbent as well. The synergistic effect based on both adsorption and photocatalysis is a key factor concerning a very high pollutant removal efficiency. The photoactivity under sunlight is higher than that observed under visible light, supporting the practical use of the BiOI photocatalyst for the removal of toxic pollutants by utilizing abundant solar energy [8].

**Figure 5:**
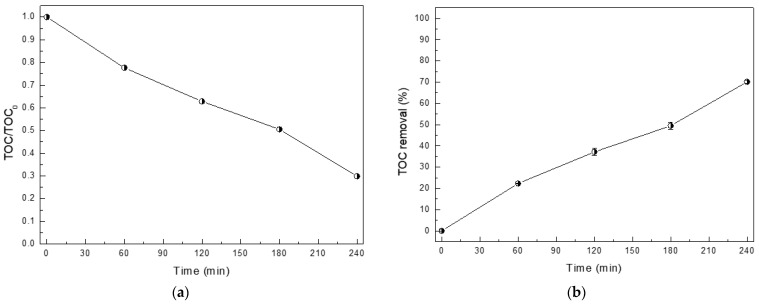
Total organic carbon (TOC) in RhB dye solution as a function of time (**a**) and the corresponding photocatalytic activity (**b**).

To confirm the mineralization of the pollutant solution, the total organic carbon (TOC) was investigated in the photodegradation of RhB dye. A decrease in the TOC content with an increase in the photo illumination time was detected (Figure 5a), implying the mineralization of the RhB dye by the BiOI photocatalyst under photo illumination. A performance of 70% was observed after 240 min of light irradiation (Figure 5b). The TOC result confirmed the oxidation of the RhB dye to CO_2_, H_2_O, and some small molecules. The result agrees well with those reported previously in the literature regarding the photodegradation of organic dyes in the presence of either ZnO or BiOCl photocatalysts [8,24].

**Figure 6:**
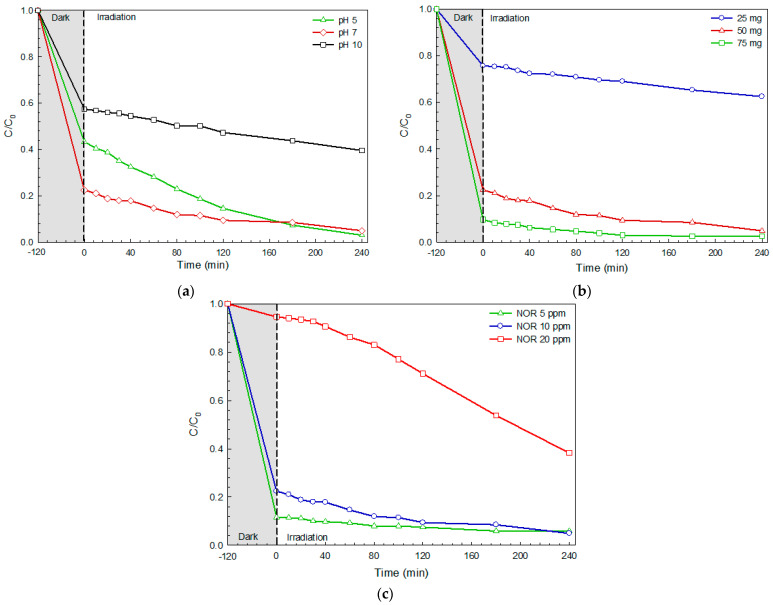
The effect of initial solution pH (**a**), photocatalyst content (**b**), and dye concentration (**c**) on the efficiency of the degradation of RhB dye.

The effect of some parameters including the initial concentration, photocatalyst content, and initial solution pH on the photodegradation of Rhodamine B (RhB dye) was elucidated [3]. In principle, pH is one important factor affecting photocatalytic performance. It has been reported that changes in pH remarkably affected the photodegradation rate of an organic pollutant [8,24,28]. Thus, the influence of the solution pH on the degradation of RhB dye was investigated. A pH of about 7 is the natural pH of RhB dye solution. The BiOI photocatalyst exhibited the greatest photocatalytic activity at the natural pH (Figure 6a). In the basic solution of pH = 10, however, a negative charge on the BiOI photocatalyst surface and on the RhB dye molecules was seen to occur. Therefore, the repulsion between the photocatalyst and RhB dye lowers the RhB dye adsorption on the photocatalyst surface, which lowers the resultant photocatalytic performance. In contrast, by using an acidic solution of pH = 5, a decrease in photocatalytic efficiency was detected. This is attributed to the dissolution of the BiOI photocatalyst in acidic conditions [3,8]. Thus, further work was carried out by controlling the initial solution pH at the natural pH of RhB dye.

The effect of the photocatalyst content (25–75 mg) on the photoactivity of the photocatalyst was also investigated (Figure 6b). Increasing the photocatalyst content resulted in an enhancement of the photocatalytic efficiency. This may be attributed to the increase in the number of RhB molecules adsorbed on the photocatalyst surface together with increments in the photocatalyst particle density per unit area of light illumination [3,8,24,28]. Incorporation of photocatalyst content from 25 mg to 50 mg caused a remarkable improvement in the photocatalytic activity. It should be noted that the addition of the photocatalyst content to up to 75 mg did not strongly increase the performance of the photocatalyst. Therefore, the photocatalyst of 50 mg was selected for further investigation.

The influence of the initial concentration on performance was also elucidated. An increase in RhB dye concentration resulted in a decrease in photoactivity (Figure 6c). Upon increasing the RhB concentration, most of the light could be absorbed by the RhB dye instead of the BiOI photocatalyst. Thus, a decrease in photo flux entering the surface of the photocatalyst would occur. This would cause a decrease in the performance of the photocatalyst [3,8]. It can be clearly seen that the greatest RhB concentration of 20 ppm provided the lowest photocatalytic performance.

#### 3.2.2. Photodegradation Mechanism and Cycling Ability

**Figure 7:**
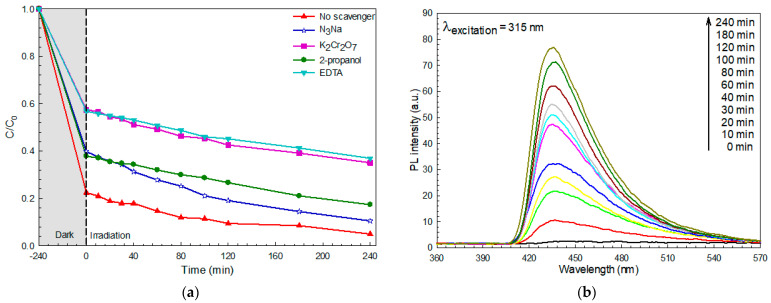
A decrease in C/C_0_ with time due to photodegradation of RhB dye after addition of various scavengers (**a**), and hydroxyl radical trapping PL spectra of the prepared BiOI photocatalyst after photo irradiation (**b**).

The photodegradation mechanism of the pollutant was proposed by determining the results obtained from the trapping experiment [3,8,24,28]. The effect of some quenchers on RhB dye removal was examined after the incorporation of 2-propanol, NaN_3_, EDTA-2Na, and K_2_Cr_2_O_7_ as a quencher of ^•^OH, ^•^O_2_^−^, *h*^+^, and *e*^−^, respectively. In the presence of either NaN_3_ or 2-propanol, the C/C_0_ was close to that obtained from the no-scavenger process (Figure 7a). A corresponding efficiency of higher than 80% was observed after the incorporation of either NaN_3_ or 2-propanol. On the contrary, a dramatic lowering of the performance to nearly 40% was detected after the incorporation of either K_2_Cr_2_O_7_ or EDTA-2Na. The results indicate the vital role of both photogenerated electrons and holes in the removal of RhB dye in the solution.

Confirmation of the generated hydroxyl radicals (^•^OH), after photo irradiation, was performed by studying the terephthalic acid (TA) trapping experiment [3,28]. In principle, the reaction of TA and hydroxyl radicals should result in the formation of a highly fluorescent 2-hydroxyterephthalic acid (HTA). The addition of PL intensity at 425 nm after increasing the light illumination time was observed (Figure 7b), suggesting the creation of HTA. The result indicates that radicals play a role in the removal of pollutants.

After photo irradiation of the semiconducting photocatalyst, in principle, the generation of the electron and hole was achieved in the conduction band (CB) and the valence band (VB), respectively. After that, the reactive species were generated. The level of the CB and the VB potentials of the BiOI photocatalyst can be evaluated by using the Mulliken electronegativity theory [3,28], as shown below.

*E*_VB_ = *χ* − *E*_C_ + 0.5*E*_g_ (4)

*E*_CB_ = *E*_VB_ − *E*_g_ (5)

where *E*_VB_ and *E*_CB_ are the VB potential and CB potential, respectively. *E*_C_ and *χ* represents the standard hydrogen electrode potential (≈4.5 eV) and the absolute value of the electronegativity (6.65 eV) of the prepared BiOI, respectively [18]. The corresponding VB and CB level of 2.78 and 0.63 eV, respectively, can be obtained from the prepared BiOI photocatalyst. The degradation mechanism of the pollutants can be proposed as follows.

BiOI + *hν* → BiOI + *e*^−^ + *h*^+^ (6)

*e*^−^ + O_2_ → ^•^O_2_^−^ (7)

^•^O_2_^−^+ 2H_2_O + *e*^−^ → 2^•^OH + 2OH^−^ (8)

*h*^+^ + OH^−^ → ^•^OH (9)

^•^OH + pollutants → products (10)

*h*^+^ + pollutants → products (11)

**Figure 8:**
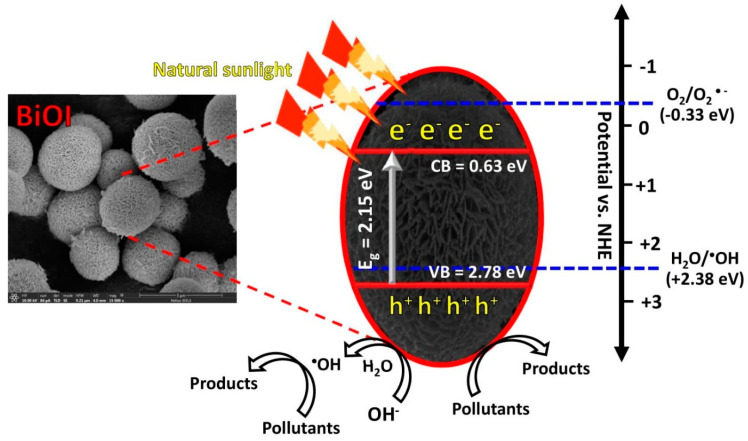
A possible photodegradation mechanism of organic pollutants over BiOI photocatalyst after photo illumination.

**Figure 9:**
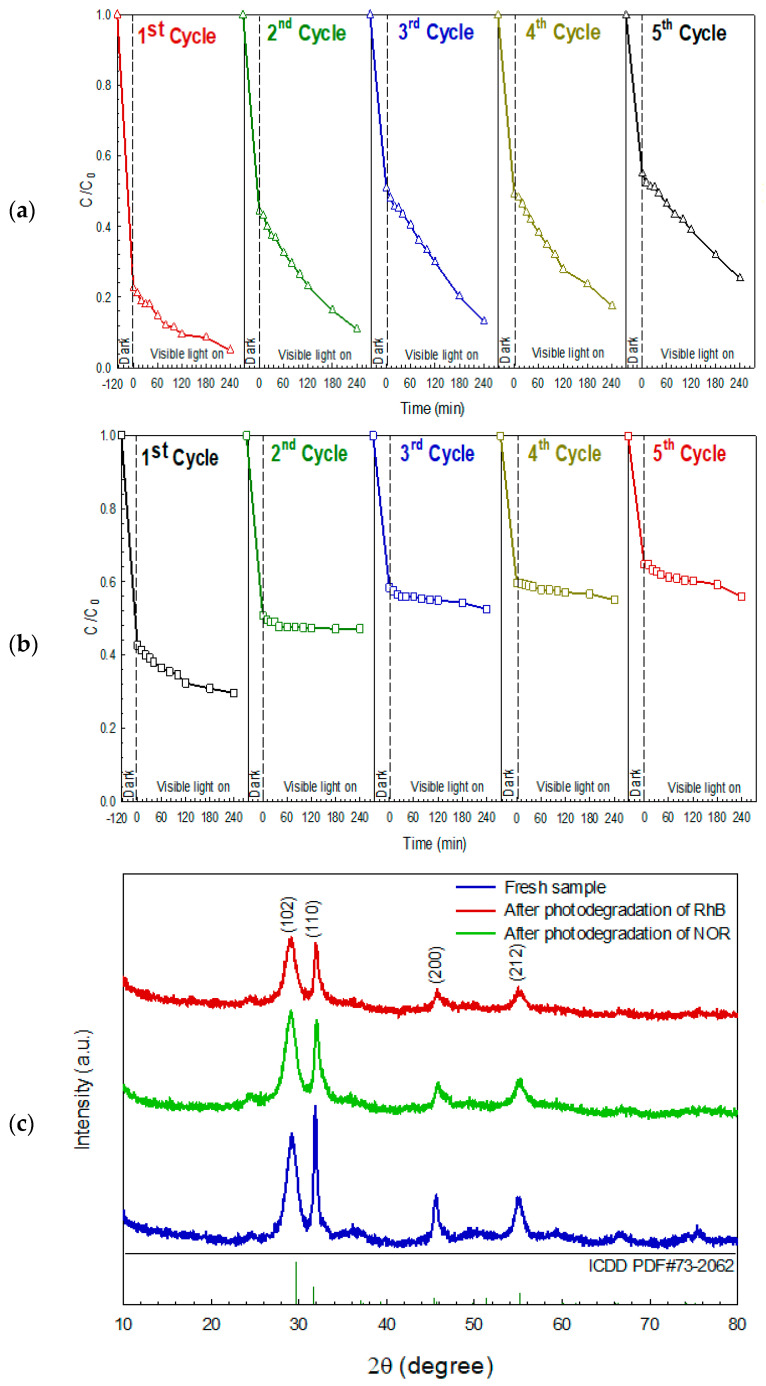

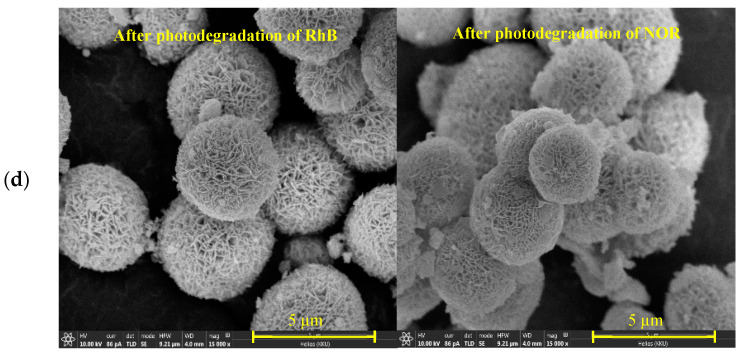
Reusability of the photocatalyst in the degradation of RhB dye (**a**), and NOR antibiotic (**b**), XRD patterns (**c**), and SEM micrographs (**d**) after the photodegradation of RhB dye and NOR antibiotic.

The detail of the photocatalytic degradation mechanism of the organic pollutants by the prepared BiOI photocatalyst can be summarized as shown in Figure 8. The stability of the sample is a crucial factor concerning its real-scale utilization. The cycling ability of the BiOI after the removal of organic pollutants was elucidated [3,8,24,28]. After five times uses, the BiOI photocatalyst retains its promising performance toward degradation of RhB and NOR (Figure 9a,b). The structural stability of the BiOI photocatalyst was also investigated. The XRD patterns (Figure 9c) of the photocatalyst from both before and after pollutant degradation were similar, implying the structural stability of the sample. Moreover, the morphology of the BiOI photocatalyst after the photodegradation study (Figure 9d) was identical to that of the fresh photocatalyst (Figure 2a). This also strongly supports the morphological stability of the BiOI photocatalyst [3,24,28,29,30,31,32].

The photoactivity of the photocatalysts in the photodegradation of various organic pollutants has been investigated [3,24,28,33,34,35,36,37]. As seen in Table S1 (Supplementary Materials), various photocatalysts including binary and ternary composites showed an approximately 70–97% removal of NOR antibiotics. In addition, the degradation of RhB dye in the range of 92–100% can be obtained from numerous photocatalysts. Herein, the BiOI photocatalyst has been utilized for the removal of both dye and antibiotic pollutants. The solvothermally grown BiOI provided visible-light-responsive photoactivity of 71% and 95% toward the degradation of RhB and NOR, respectively. Interestingly, the prepared BiOI photocatalyst also provided a promising sunlight-active efficiency of 100% without the addition of a capping agent or the creation of composites. The present work demonstrates the enhanced photocatalytic performance of the prepared BiOI photocatalyst for the removal of pollutants by using abundant solar energy.

## 4. Conclusions

A solvothermally grown tetragonal BiOI photocatalyst with a band gap of 2.15 eV was prepared using a green method. The catalyst provides a high sunlight-active photoactivity of 100% in the photodegradation of RhB dye due to its great charge carrier separation efficiency at the interface. The degradation reaction correlates well with the first-order reaction. The photogenerated hole and electron play major roles in the removal of the pollutant. The prepared BiOI catalyst remains stable and still exhibits promising efficiency after five runs, indicating its enhanced efficiency for the detoxification of pollutants in wastewater. This is due to the very high surface area (about 118 m^2^ g^−1^) of the prepared BiOI, which can also act as an excellent adsorbent. The contribution of both adsorption and photocatalysis is a major factor concerning very high pollutant-removal efficiency. Enhanced performance under natural solar light supports the practical use of the BiOI photocatalyst for the removal of toxic organic pollutants in aqueous solutions by the utilization of abundant solar energy.

## Data Availability

Not applicable.

## Supplementary Materials

The following are available online, Table S1: Comparison of pollutant degradation by using various photocatalysts.

## Sample Availability

Samples of the compounds are not available from the authors.
