# Supplementary material for: Sunlight-Active BiOI Photocatalyst as an Efficient Adsorbent for the Removal of Organic Dyes and Antibiotics from Aqueous Solutions
Source: Molecules. 2021 Sep 16;26(18):5624. doi: 10.3390/molecules26185624 (PMC8471800; doi:10.3390/molecules26185624)
Supplement: Supplementary file 1 [file molecules-26-05624-s001.zip › molecules-1374867-supplementary.pdf]

Supplementary Materials

Sunlight-Active BiOI Photocatalyst as an Efficient Adsorbent for the Removal of Organic Dyes and Antibiotics from Aqueous Solutions

Teerapong Narenuch, Teeradech Senasu, Tammanoon Chankhanittha and Suwat Nanan*

Materials Chemistry Research Center, Department of Chemistry and Center of Excellence for Innovation in Chemistry (PERCH-CIC), Faculty of Science, Khon Kaen University, Khon Kaen 40002, Thailand
* Correspondence: suwatna@kku.ac.th; Tel: +66-43-202222-41 (ext. 12370); Fax: +66-43-202373

Table S1. Comparison of pollutant degradation by using various photocatalysts

Photocatalyst	Pollutants	Conc.	Catalyst loading	Light source	Lamp	Time (min)	Degradation (%)	Ref.
BiOBr	NOR	20 mgL^{-1}	50 mg	-	-	180	97.2	[1]
BiOBr-Bi	NOR	20 mgL^{-1}	50 mg	Visible	500 W xenon lamp	180	97.2	[2]
Bi/BiOBr/CdS	NOR	20 mgL^{-1}	50 mg	Visible	500 W xenon lamp	180	97.4	[3]
BiOBr/UiO-66-NH_2	NOR	10 mgL^{-1}	15 mg	Visible	500 W xenon lamp	180	93.6	[4]
$CoFe_2O_4$-rGO-BiOBr	NOR	5 mgL^{-1}	25 mg	Visible	Mercury lamp	40	88.7	[5]
BiOBr/β-Bi_2O_3	NOR	20 mgL^{-1}	50 mg	Solar	500 W xenon lamp	60	70.0	[6]
BiOI	NOR	10 mgL^{-1}	50 mg	Sunlight	-	240	78.1	This work
WO_3	RhB	40 mgL^{-1}	50 mg	Visible	500 W xenon lamp	100	92.8	[7]
$Cs_3PW_{12}O_{40}/WO_3$	RhB	40 mgL^{-1}	60 mg	Visible	55 W xenon lamp	30	100	[8]
$Ag/Ag_3PO_4/WO_3$	RhB	10 mgL^{-1}	20 mg	Visible	300 W xenon lamp	120	100	[9]
CdS/AgBr-rGO	RhB	5 mgL^{-1}	60 mg	Visible	500 W xenon lamp	60	95.8	[10]
Ag/AgBr/ZnO	RhB	5 mgL^{-1}	100 mg	Visible	300 W Tungsten lamp	180	100	[11]
BiOI	RhB	10 mgL^{-1}	50 mg	Sunlight	-	60	100	This work

References

[1] Z. Gao, B. Yao, T. Xu, Comparative study on photocatalytic material activity of BiOBr flower microspheres and sheet structure, Environ. Technol. (2019) 1–11. https://doi.org/10.1080/09593330.2019.1670268.

[2] Z. Gao, B. Yao, F. Yang, T. Xu, Y. He, Preparation of BiOBr-Bi heterojunction composites with enhanced photocatalytic properties on BiOBr surface by in-situ reduction, Mater. Sci. Semicond. Process. 108 (2020) 104882. https://doi.org/10.1016/j.mssp.2019.104882.

[3] Z. Gao, B. Yao, T. Xu, M. Ma, Effect and study of reducing agent $NaBH_4$ on Bi/BiOBr/CdS photocatalyst, Mater. Lett. 259 (2020) 126874. https://doi.org/10.1016/j.matlet.2019.126874.

[4] Z. Yang, X. Tong, J. Feng, S. He, M. Fu, X. Niu, T. Zhang, H. Liang, A. Ding, X. Feng, Flower-like BiOBr/UiO-66-NH_2 nanosphere with improved photocatalytic property for norfloxacin removal, Chemosphere. 220 (2019) 98–106. https://doi.org/10.1016/J.CHEMOSPHERE.2018.12.086.

[5] R. Zhang, Y. Li, Q. Han, T. Zhang, Y. Liu, K. Zeng, C. Zhao, Investigation the High Photocatalytic Activity of Magnetically Separable Graphene Oxide Modified BiOBr Nanocomposites for Degradation of Organic Pollutants and Antibiotic, J. Inorg. Organomet. Polym. Mater. 30 (2020) 1703–1715. https://doi.org/10.1007/s10904-019-01333-7.

[6] M. Xu, M. Ye, X. Zhou, J. Cheng, C. Huang, W. Wong, Z. Wang, Y. Wang, C. Li, One-pot controllable synthesis of BiOBr/β-Bi_2O_3 nanocomposites with enhanced photocatalytic degradation of norfloxacin under simulated solar irradiation, J. Alloys Compd. 816 (2020) 152664. https://doi.org/10.1016/J.JALLCOM.2019.152664.

[7] G. Palanisamy, K. Bhuvaneswari, T. Pazhanivel, G. Bharathi, Enriched photocatalytic activity of Rhodamine B dye from aqueous solution using hollow sphere tungsten trioxide

[8] N. Tahmasebi, S. Mirzavand, A. Hakimyfard, S. Barzegar, The excellent photocatalytic activity of novel $Cs_3PW_{12}O_{40}/WO_3$ composite toward the degradation of rhodamine B, Adv. Powder Technol. 30 (2019) 257–265.

[10] J. Zhang, Z. Zhang, W. Zhu, X. Meng, Boosted photocatalytic degradation of Rhodamine B pollutants with Z-scheme CdS/AgBr-rGO nanocomposite, Appl. Surf. Sci. 502 (2020) 144275.

[9] Q. Li, F. Wang, Y. Hua, Y. Luo, X. Liu, G. Duan, X. Yang, Deposition-precipitation preparation of $Ag/Ag_3PO_4/WO_3$ nanocomposites for efficient Visible-light degradation of rhodamine B under strongly acidic/alkaline conditions, J. Colloid Interface Sci. 506 (2017) 207–216.

[11] L. Shi, L. Liang, J. Ma, Y. Meng, S. Zhong, F. Wang, J. Sun, Highly efficient visible light-driven Ag/AgBr/ZnO composite photocatalyst for degrading Rhodamine B, Ceram. Int. 40 (2014) 3495–3502.
